# Item-based analysis of the effects of duloxetine in depression: a patient-level post hoc study

**DOI:** 10.1038/s41386-019-0523-4

**Published:** 2019-09-14

**Authors:** Alexander Lisinski, Fredrik Hieronymus, Jakob Näslund, Staffan Nilsson, Elias Eriksson

**Affiliations:** 1 Department of Pharmacology, Institute of Neuroscience and Physiology, Sahlgrenska Academy, University of Gothenburg, Gothenburg, Sweden; 2 Department of Pathology and Genetics, Institute of Biomedicine, Sahlgrenska Academy, University of Gothenburg, Gothenburg, Sweden; 3 Institute of Mathematical Sciences, Chalmers University of Technology, Gothenburg, Sweden

**Keywords:** Depression, Outcomes research

## Abstract

Oft-cited trial-level meta-analyses casting doubt on the usefulness of antidepressants have been based on re-analyses of to what extent the active drug has outperformed placebo in reducing the sum score of the Hamilton Depression Rating Scale (HDRS-17-sum) in clinical trials. Recent studies, however, suggest patient-level analyses of individual HDRS items to be more informative when assessing the efficacy of an antidepressant. To shed further light on both symptom-reducing and symptom-aggravating effects of a serotonin and noradrenaline reuptake inhibitor, duloxetine, when used for major depression in adults, we hence applied this approach to re-analyse data from 13 placebo-controlled trials. In addition, using patient-level data from 28 placebo-controlled trials of selective serotonin reuptake inhibitors (SSRIs), the response profile of duloxetine was compared to that of these drugs. Duloxetine induced a robust reduction in depressed mood that was not dependent on baseline severity and not caused by side-effects breaking the blind. A beneficial effect on depressed mood was at hand already after one week; when outcome was assessed using HDRS-17-sum as effect parameter, this early response was however masked by a concomitant deterioration with respect to adverse event-related items. No support for a suicide-provoking effect of duloxetine was obtained. The response profile of duloxetine was strikingly similar to that of the SSRIs. We conclude that the use of HDRS-17-sum as effect parameter underestimates the true efficacy and masks an early effect of duloxetine on core symptoms of depression. No support for major differences between duloxetine and SSRIs in clinical profile were obtained.

## Introduction

The ongoing debate regarding the alleged ineffectiveness of antidepressants has to a large extent been based on trial-based meta-analyses using the sum score of the 17-item Hamilton Depression Rating Scale (HDRS-17-sum) as effect parameter [1]. The HDRS-17 has, however, been criticised for shortcomings likely to reduce the apparent difference between active drug and placebo: it is multidimensional, it includes items that are often absent at baseline, it includes non-specific symptoms often present also in non-depressed patients, and it may be contaminated by common antidepressant side effects [2–6]. Thus, in patient-level analyses comparing selective serotonin reuptake inhibitors (SSRIs) to placebo with respect to reduction in the first item of the HDRS-17, depressed mood, a considerably more consistent and robust superiority of active drug over placebo was revealed as compared to when HDRS-17-sum was used as effect parameter [5, 6].

One purpose of the present work was to use patient-level data to explore if these results can be extended to an antidepressant with a somewhat different pharmacological profile, the serotonin and noradrenalin reuptake inhibitor (SNRI) duloxetine [7]. Prompted by claims that a possible effect of antidepressants be restricted to the most severe cases [1, 8], or be merely the consequence of side effect breaking the blind in patients [9], these possibilities were also addressed, as was the issue of to what extent duloxetine may cause initial anxiety- or suicide-provoking effects. Whereas some of these aspects have been examined in previous meta-and mega-analyses [10–12], this is, to our knowledge, the most comprehensive patient-level post hoc analysis of duloxetine to date. Likewise, it is the most extensive analysis yet undertaken regarding possible differences between duloxetine and the SSRIs with respect to response profile.

## Materials and methods

### Data acquisition and participants

Patient-level data for 15 drug company-sponsored, placebo-controlled clinical trials regarding the treatment of depression in adults with duloxetine and using the HDRS-17 for assessment of efficacy were obtained from the Clinical Study Data Request (CSDR) website; whereas 10 of these were named in the FDA Approval Packages for duloxetine, five were post-marketing trials. We confirmed that we had access to all relevant trials by searching the FDA Approval Packages, the CSDR website, Eli Lilly’s Clinical Study Results portal and Clinicaltrials.gov. For one trial named in the FDA Approval Package, HMAG, data were not available. For two of the trials, HMAH and HMAI, we only had access to information regarding HDRS-17-sum and the items depressed mood and suicidality; moreover, since these trials only included sub-therapeutic doses (5–20 mg), they were included in a sensitivity analysis only. While all remaining trials included adult patients aged at least 18, two of them, HMBV and HMFA, included elderly patients only and one, HMCB, only patients scoring a minimum of two points on the Brief Pain Inventory scale. For the comparison of duloxetine and the SSRIs with respect to item response profile, we also used patient-level data from 28 drug company-sponsored placebo-controlled trials of citalopram, paroxetine and sertraline; for details regarding this database, see ref. [5]. Since some duloxetine studies were so designed that neither patients nor investigators knew at which visit the administration of active treatment started (double-blind variable lead-in),[13] 486 patients displayed HDRS-17 ratings below 15 points at the start of active treatment. These were all retrospectively excluded by the investigators in the original studies [14–20], and so also in our primary analyses. In total, our primary analyses comprised 3575 subjects.

### Statistical analysis

Linear mixed models including change score for the relevant measure (HDRS-17-sum or individual items) as the dependent variable, time (week), treatment (duloxetine or placebo), trial and the treatment*time interaction as fixed factors, and baseline rating for the corresponding outcome measure as covariate, were used to assess the effect of treatment (duloxetine) on all included outcome measures. For mean scores of the suicidality item, the analysis comprising all subjects was complemented with analyses undertaken after splitting the cohort into those 18–24 and those ≥25 years of age, respectively. The model described above was expanded when assessing the possible interaction between HDRS-17-rated baseline severity and treatment and when comparing the relative efficacy of duloxetine to that of the SSRIs (see below). ANCOVA models on the observed cases population were used when addressing the association between side effects and treatment efficacy (see below).

For all linear mixed models, an unstructured covariance matrix was used to model within-patient errors; if the model did not converge, an autoregressive heterogeneous structure was used instead. Denominator degrees of freedom were estimated using the Kenward-Roger approximation. Effect sizes were calculated by dividing the least-squares mean differences for the relevant contrast by the root of the variance for the corresponding time point.

The possible relationship between baseline severity measured by HDRS-17-sum and outcome was assessed using a model similar to the one described above, but always including the baseline score for HDRS-17-sum (rather than the baseline score of the dependent variable) as well as the corresponding two-way (baseline score*treatment, baseline score*time) and three-way (baseline score*treatment*time) interactions. HDRS-17 sum, depressed mood, the sum of six core symptoms of depression (HDRS-6) [21] and the sum of the remaining 11 symptoms (non-HDRS-6) served as effect parameters [22]. The outcome of interest was the parameter estimates for the two-way interaction between HDRS-17-sum rated baseline severity and treatment since this parameter corresponds to the interaction effect at the endpoint visit (week 8), which was the reference category. In addition, patients at the extreme ends of the severity range, scoring ≤18 and ≥27 respectively, i.e. the lowest proposed cut-off for moderate depression and the highest proposed for severe depression [23], were compared with respect to baseline symptomatology and with respect to endpoint effect sizes for HDRS-6, non-HDRS-6 and all individual items.

Since a possible association between side effects and outcome may be masked by patients with adverse events more often discontinuing early, and by potential side effects of antidepressants contaminating the HDRS-17-sum measure, the analysis addressing this issue comprised completers only and used depressed mood as effect parameter; a sensitivity analysis replacing depressed mood with HDRS-17-sum is, however, also presented. Presence of adverse events either during the first 2 weeks of treatment, or at any time until endpoint, was coded as a dichotomous variable (yes/no) [24]. Adverse events that had been reported already before the initiation of treatment were excluded. The three groups thus compared, using analysis of covariance (ANCOVA) with baseline values on the corresponding outcome measure as covariate and trial as fixed factor, were placebo-treated patients, duloxetine-treated patients with side effects and duloxetine-treated patients without side effects. For assessing the possible association between adverse event severity and reduction in depressed mood, only duloxetine-treated patients with adverse events were included, with the highest side effect severity, coded as mild, moderate or severe, being included in an ANCOVA as described above.

Finally, the item response profile of duloxetine was compared to that of the SSRIs. To this end, patient-level data from the duloxetine trials on the one hand, and patient-level data from 28 relevant drug company-sponsored trials conducted for citalopram, paroxetine and sertraline [5] on the other, were used to assess the relative effect size for each HDRS item using the sum of all other HDRS items as covariate in two separate linear mixed models. For these analyses week 6 served as endpoint since many of the SSRI studies had been of only six weeks duration. In those of the SSRI trials in which fluoxetine had been used as an active comparator, this treatment arm was also included in the analyses. Student’s *t*-test was used to compare subjects treated with duloxetine and an SSRI, respectively, with regard to the relative mean active drug versus placebo differences.

For all analyses, significance tests were two-tailed with an alpha level of .05. No correction for multiple testing was undertaken.

Remote desktop access to the Clinical Trial Data Transparency environment was provided by CSDR through SAS Solutions OnDemand, using SAS version 9.4 (SAS Institute, Cary, NC, USA).

### Sensitivity analyses

To assess if the decision to exclude two low-dose trials impacted the outcome, sensitivity analyses comprising all doses were undertaken. Similarly, to explore the possible influence of excluding patients scoring <15 points on the HDRS-17 at baseline, sensitivity analyses including these patients were performed. A final sensitivity analysis addressed the possible impact of side effects on outcome using the HDRS-17-sum rather than depressed mood as effect parameter. For all sensitivity analyses, the same statistical models as in the corresponding main analysis were used.

### Ethics

The Regional Ethics Review Board of Gothenburg, Sweden, issued an advisory opinion stating no objection to the conduct of this study.

## Results

### Effect sizes for individual items

**Fig. 1:**
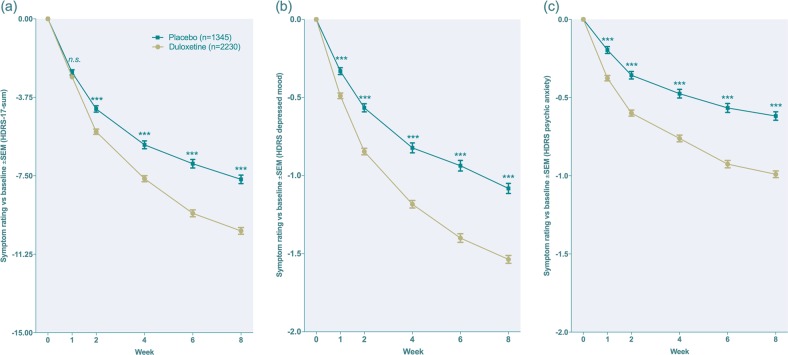
**a**–**c** Week-by-week mean change for HDRS-17-sum (**a**), the depressed mood item (**b**) and the psychic anxiety item (**c**) for duloxetine- and placebo-treated subjects, respectively. Lines represent estimated means from a linear mixed model. Effect sizes at week 1, 2, 4, 6 and 8, respectively: **a** 0.05, 0.20, 0.27, 0.37, 0.37; **b** 0.20, 0.31, 0.37, 0.46, 0.44 **c**; 0.23, 0.29, 0.34, 0.42, 0.43. *n.s.* non-significant (*p* = 0.16); *** = *p* < 0.001

**Fig. 2:**
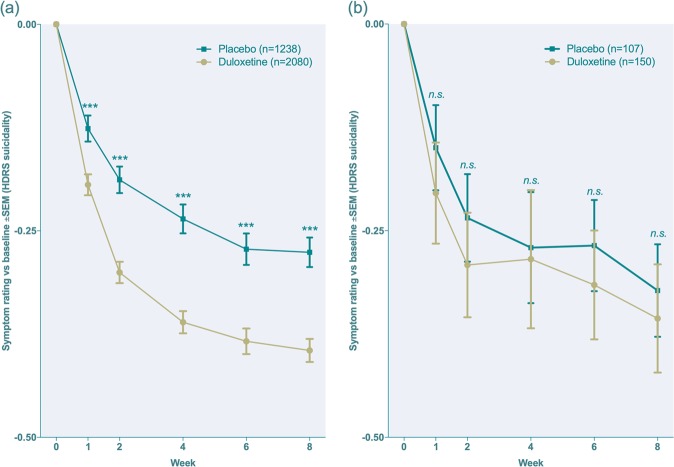
**a,** **b** Week-by-week mean change for the suicidality item for duloxetine and placebo-treated subjects, respectively, in subjects aged 25 years and above (**a**) subjects aged 18–24 years (**b**). Lines represent estimated means from a linear mixed model. Baseline scores **a** 0.62 points, standard deviation (SD) 0.76; **b** 0.53 points, SD 0.70. Effect sizes at week 1, 2, 4, 6 and 8, respectively: **a** 0.15, 0.23, 0.26, 0.23, 0.24; **b** 0.22, 0.22, 0.13, 0.25, 0.20. *n.s.* non-significant (*p* = 0.10, 0.11, 0.42, 0.16, 0.21); ***p < 0.001

Baseline characteristics for all included trials are displayed in Supplementary table 1. Mean values of HDRS-17-sum and depressed mood at baseline were 21.3 points (duloxetine: 21.3 points, placebo: 21.3 points) and 2.71 points (duloxetine: 2.71 points, placebo: 2.69 points), respectively. Effect sizes and levels of significance for duloxetine with respect to HDRS-17-sum as well as all individual items for weeks 1, 6 and 8 are presented in Table 1. Three studies however lacked an evaluation at week 6 and 8; for these observations, week 7 (HMCB) or week 9 (HMBHa + b) were used as endpoint observations, and week 5 (HMCB) or 7 (HMBHa + b) as replacement for week 6 data. Week-for-week data for HDRS-17-sum, depressed mood and psychic anxiety are presented in Fig. 1a–c and for suicidality in Fig. 2a, b.

**Table 1:** Baseline means, effect sizes and p-values for HDRS-17-sum and individual items

Measure of efficacy (scoring range)	Baseline mean	*s.d*.	ES	*p*-value	ES	*p*-value	ES	*p*-value
			Week 1		Week 6		Week 8	
HDRS-17-sum	21.3	3.9	0.05	0.16	0.37	<0.001	0.37	<0.001
Depressed mood (0–4)	2.7	0.6	0.20	<0.001	0.46	<0.001	0.44	<0.001
Feelings of guilt (0–4)	1.6	0.8	0.10	0.004	0.27	<0.001	0.28	<0.001
Suicidal ideation (0–4)	0.6	0.8	0.15	<0.001	0.22	<0.001	0.23	<0.001
Insomnia, early (0–2)	1.2	0.8	0.05	0.14	0.06	0.18	0.09	0.02
Insomnia, middle (0–2)	1.3	0.8	−0.08	0.02	0.03	0.45	0.03	0.52
Insomnia, late (0–2)	1.1	0.8	−0.02	0.60	0.18	<0.001	0.13	0.001
Work & activities (0–4)	2.7	0.6	0.05	0.13	0.30	<0.001	0.32	<0.001
Retardation (0–4)	1.0	0.8	0.04	0.29	0.29	<0.001	0.22	<0.001
Agitation (0–4)	0.9	0.9	0.08	0.02	0.11	0.01	0.10	0.01
Anxiety, psychic (0–4)	2.0	0.8	0.23	<0.001	0.42	<0.001	0.43	<0.001
Anxiety, somatic (0–4)	1.5	0.8	-0.04	0.25	0.09	0.04	0.13	0.001
Somatic symptoms, gastrointestinal (0–2)	0.6	0.6	-0.30	<0.001	0.08	0.09	0.09	0.03
Somatic symptoms, general (0–2)	1.6	0.6	−0.02	0.66	0.25	<0.001	0.19	<0.001
Genital symptoms (0–2)	1.2	0.8	−0.10	0.004	0.05	0.29	0.10	0.01
Hypochondriasis (0–4)	1.0	0.9	0.04	0.27	0.22	<0.001	0.22	<0.001
Loss of weight (0–2)	0.2	0.5	−0.22	<0.001	−0.03	0.55	−0.05	0.19
Insight (0–2)	0.1	0.4	−0.05	0.15	0.04	0.34	0.07	0.07

*s.d.* standard deviation, *ES* effect size

The effect size for the comparison of duloxetine with placebo was higher for depressed mood and psychic anxiety than for HDRS-17-sum at all time points (Fig. 1a–c). A significant superiority of duloxetine over placebo was found for the items depressed mood, guilt, suicidality, agitation and psychic anxiety, but not for HDRS-17-sum, at week one. Ratings of the items middle insomnia, gastrointestinal symptoms, sexual dysfunction and loss of weight were higher in duloxetine-treated patients after one week of treatment but not at endpoint (Table 1). Analyses of the rating of suicidality after splitting the population into those 18–24 or ≥25 years of age, respectively, yielded a significant superiority of duloxetine from week one and onwards in those ≥ 25 years of age (*n* = 3 318). In those 18–24 years of age (*n* = 257), patients on duloxetine displayed numerically but non-significantly lower ratings (Fig. 2a, b).

### Relation between initial severity and outcome

The interaction between baseline severity based on HDRS-17-sum and treatment was non-significant when using the HDRS-17-sum (beta 0.10, standard error of the mean (SEM) 0.07, *p* *=* 0.11), depressed mood (beta 0.01, SEM 0.01, *p* = 0.56) or the sum of 6 core symptoms of depression (beta 0.02, SEM 0.04, *p* *=* *0*.*50*) as outcome measure, but significant in the direction of larger drug-placebo differences with increasing severity when analysing the sum of the remaining 11 symptoms (beta 0.08, SEM 0.03, *p* *=* 0.02). Baseline symptomatology and endpoint effect sizes for the extreme severity groups (≤18 and ≥27, respectively) are presented in Supplementary Table 2 and Supplementary Fig. 1a, b. While the mean non-HDRS-6 sum score was 139% higher in the high severity group, the HDRS-6 sum score was merely 63% higher. Likewise, while the effect sizes for the HDRS-6 sum score and for individual items comprising this subscale were similar in the two extreme severity groups, the effect sizes for the sum score of the non-HDRS-6 items, and for several of the items within this subscale, were notably lower in the non-severe group.

### Relation between side effects and outcome

**Fig. 3:**
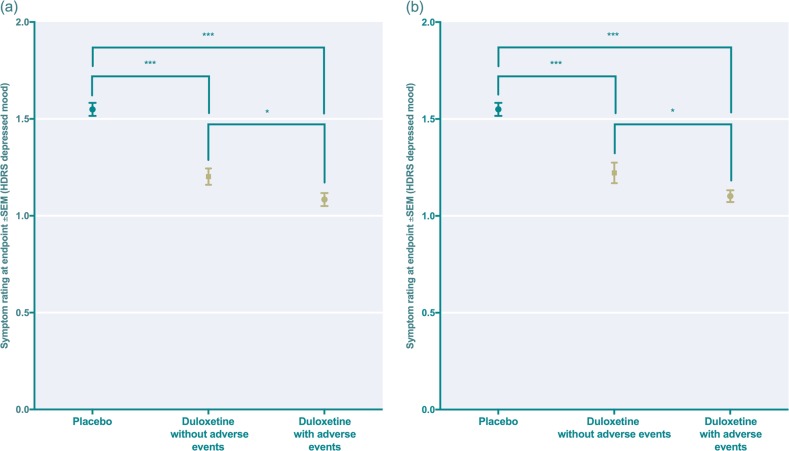
**a**, **b** Estimated endpoint means and effect sizes for the depressed mood item in placebo-treated subjects and in duloxetine-treated subjects with or without adverse events **a** only counting initial adverse events (reported during week 1–2 and **b** counting any adverse event throughout the trial. Placebo: *n* *=* 998; duloxetine without early adverse events: *n* *=* 713; duloxetine with early adverse events: *n* *=* 1 029; duloxetine without any adverse event throughout the trial: *n* *=* 449; duloxetine with any adverse event throughout the trial: *n* *=* 1 293. Effect sizes: **a** duloxetine with adverse events vs placebo: 0.46; duloxetine without adverse events vs placebo: 0.34, duloxetine with adverse events vs duloxetine without adverse events: 0.12 **b** duloxetine with adverse events vs placebo: 0.44: duloxetine without adverse events vs placebo: 0.36; duloxetine with adverse events vs duloxetine without adverse events: 0.12. *p = 0.02 (**a**), *p* = 0.04 (**b**); ***p < 0.001

Both duloxetine-treated patients not reporting early side effects and those not reporting any side effects throughout the trial displayed a significant reduction in depressed mood when compared to those treated with placebo (Fig. 3a, b). This response was moderately but significantly larger in those with side effects, but there was no impact of side effect severity on outcome (Supplementary Fig. 2).

### Symptom response patterns in patients treated with duloxetine or SSRIs

**Fig. 4:**
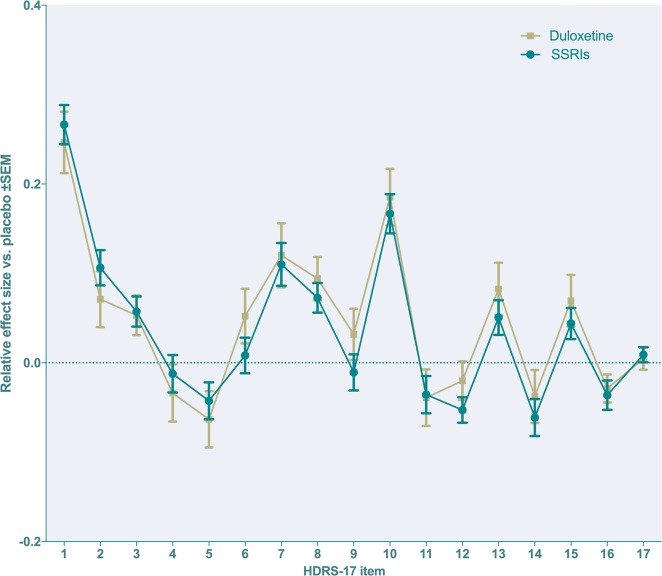
Relative effect sizes versus placebo for duloxetine and four SSRIs (grouped) using the sum of all other HDRS-17 items as covariate. Placebo (duloxetine studies): *n* *=* 1 345; duloxetine: *n* = 2 230; placebo (SSRI studies): *n* *=* 2 581; SSRIs: *n* = 5 681 (citalopram *n* *=* 744; fluoxetine *n* *=* 754; paroxetine *n* *=* 2 981; sertraline *n* *=* 1 202). HDRS-17 items and *p*-values for the contrast duloxetine vs SSRIs: 1 = depressed mood (*p* = 0.63), 2 = feelings of guilt (*p* = 0.35), 3 = suicidality (*p* = 0.89), 4 = early insomnia (*p* = 0.59), 5 = middle insomnia (*p* = 0.59), 6 = late insomnia (*p* = 0.24), 7 = work & activities (*p* = 0.82), 8 = psychomotor retardation (*p* = 0.49), 9 = psychomotor agitation (*p* = 0.26), 10 = psychic anxiety (*p* = 0.65), 11 = somatic anxiety (*p* = 0.93), 12 = gastrointestinal symptoms (*p* = 0.23), 13 = general somatic symptoms (*p* = 0.39), 14 = sexual dysfunction (*p* = 0.54), 15 = hypochondriasis (*p* = 0.45), 16 = weight change (*p* = 0.80) and 17 = insight (*p* = 0.78)

A comparison of all duloxetine-treated subjects with SSRI-treated patients within the development programs for citalopram, paroxetine and sertraline with respect to the relative reduction of individual items after adjusting for overall response revealed strikingly similar profiles, with no significant differences between the groups (Fig. 4).

### Sensitivity analyses

The outcome of the sensivity analyses for item-wise comparisons of duloxetine versus placebo including also low-dose studies or including all patients regardless of initial HDRS score were similar to that of the main analyses (Supplementary tables 3 and 4, respectively). The sensitivity analysis regarding the possible relationship between side effects and outcome when using HDRS-17-sum rather than depressed mood as outcome measure yielded no influence of side effects on the response to treatment (Supplementary Fig. 3a, b).

## Discussion

Based on trial-level meta-analyses using HDRS-17-sum as effect parameter, oft-cited authors have claimed that the effect of antidepressants is minute and clinically relevant, if at all, in cases of severe depression only [1, 8]. However, invoking results from patient-level, item-based post hoc analyses of placebo-controlled trials, we have previously suggested the apparently poor effect of the SSRIs to be an artefact caused by the use of a partly misleading effect parameter; thus, when exploring the effect of SSRIs on the depressed mood item, rather than on HDRS-17-sum, a consistent and robust superiority of SSRIs over placebo was revealed [5]. We now report the results of similar, patient-level, item-based post hoc analyses of a non-SSRI antidepressant, duloxetine, confirming this observation. Thus, in spite of other methodological problems associated with antidepressant trials, that should be expected to lead to an underrating of the efficacy of the active drug [5, 25], the effect size for the reduction of depressed mood obtained in the present analysis, 0.44, is in the medium range and well on par with that displayed by drugs used for somatic conditions [26] (and higher than that obtained for HDRS-17-sum). Moreover, the beneficial effect of duloxetine was not restricted to those with severe depression and not dependent on side effects breaking the blind. A comparison of patients treated with duloxetine and SSRIs, respectively, revealed a strikingly similar item response pattern. The outcome of sensitivity analyses including also low-dose studies or patients with low baseline scores was essentially the same as that of the main analyses.

The previous claim that antidepressants be ineffective in mild and moderate cases [1] has prompted authorities in many countries to recommend non-pharmacological treatment for non-severe depression. Our analysis, however, revealed the duloxetine-induced reduction in depressed mood and other core symptoms of depression in patients at the lower end of the severity spectrum to be largely on par with that observed in the most severe cases; in line with this, no significant interaction between baseline severity and treatment was observed when addressing outcome using these measures. For the reduction in HDRS-17-sum, however, a non-significant tendency for an influence of severity on response was observed, which can largely be explained by lower effect sizes for non-core symptoms in the non-severe group; thus, the effect size for the sum of the non-core items was indeed significantly associated with baseline severity. When interpreting this observation, it should be noted that the rating of non-core symptoms of depression, considerably more so than that of core symptoms, often was low or zero at baseline in cases defined as non-severe, hence leaving little room for improvement, but ample room for aggravation also in patients in remission should the item capture possible side effects of the given treatment. We hence suggest that the widely disseminated claim that antidepressants are useless for non-severe depression is misleading and the result of the unfortunate use of an outcome measure comprising many items that are often absent at baseline in the non-severe group. Though not comprising any interaction analyses corresponding to those here reported, a previous report from Shelton and co-workers [12], based on post hoc analyses of four duloxetine trials, was largely in line with our findings.

Another widely propagated claim aimed to cast doubt on the usefulness of antidepressants is that the superiority of active drug over placebo observed in antidepressant trials be merely a consequence of enhanced expectation of improvement due to side-effects breaking the blind [9]. The finding that the effect size for the reduction in depressed mood (unlike that for the reduction in HDRS-17-sum) was somewhat larger in duloxetine-treated patients reporting side effects than in those not reporting side effects could be regarded as support for this assumption; needless to say, this association may, however, also be the consequence of inter-individual differences in dose and/or duloxetine metabolism that, by influencing serum levels, may impact both response and side effects in the same direction. More importantly, the theory that side effects are the major factor for active treatment outperforming placebo was rebutted by the observation of a marked superiority of duloxetine over placebo also in patients reporting no adverse events. Likewise, no support was obtained for the suggestion by Kirsch that adverse event severity be positively associated with response [9]. Both the latter observations are in line with what we previously reported for two SSRIs [24].

Attempts to reveal the mechanism of action of SSRIs and SNRIs, beyond the inhibition of transmitter reuptake, have often been based on the postulate that no clinical effect is observed until the treatment has been ongoing for a number of weeks [27]. However, the failure to detect an early effect of drug treatment using the HDRS-17-sum may be due to early side-effects of treatment contaminating the rating, hence masking an early improvement with respect to core symptoms of depression such as depressed mood [5, 6]. In this vein, while there was no difference between treatment groups with respect to HDRS-17-sum at week 1, duloxetine-treated patients reported less severity with respect to several cardinal symptoms of depression already at this time point, including depressed mood, but significantly more middle insomnia, gastrointestinal symptoms, sexual dysfunction and loss of weight. We hence gained support for our previous conclusion, based on similar post hoc analyses of SSRI trials [6], that the antidepressant action of amine reuptake inhibitors, though slow to reach its maximum, does commence shortly after the onset of treatment. Our results are also in line with previous reports suggesting duloxetine to outperform placebo with respect to individual items already at week 1 [12, 27], as well as with a report on the use of venlafaxine in generalised anxiety disorder indicating early side effects to mask an early improvement with respect to certain symptoms (including anxiety) [28].

Antidepressants displaying high affinity for the serotonin transporter, such as clomipramine and the SSRIs, may cause an initial increase in anxiety in susceptible patients [29, 30]. Our previous attempt to clarify the early net effect of SSRIs on HDRS-17-assessed anxiety revealed psychic anxiety and agitation to be moderately lower, but somatic anxiety to be moderately higher, after the first week of treatment in those administered an SSRI [31]. The corresponding outcome for duloxetine was well in line with that observed for the SSRIs: a significant reduction in psychic anxiety and agitation but not in somatic anxiety at week 1 [11]. However, while the rating of somatic anxiety at week 1 was numerically higher for duloxetine-treated patients, this difference was not significant, as It was for the SSRIs. At week 6 and at endpoint, all three items were in favour of duloxetine. It should, however, be noted that a complete picture of a possible anxiety-provoking effect of an antidepressant requires an analysis also of the reporting of anxiety-related adverse events [31], particularly in patients discontinuing before the week 1 visit, and that no such assessment was included in this study.

Reports suggesting SSRIs to be suicide-provoking, and particularly so at the start of treatment [32] and in young subjects [33], have previously prompted us [34] and others [35] to address this issue by analysing HDRS-17-assessed suicidality at the week one visit following initiation of treatment after splitting the population into young adults and adults, respectively. While these previous studies have shown SSRIs [34, 35], as well as an SNRI, venlafaxine [35], to cause a net reduction in suicidality from week 1 or 2 and onwards in subjects ≥25 years of age, they have failed to demonstrate a corresponding effect in subjects below the age of 25, but also without providing any evidence of a suicidality-enhancing effect in this age group. A previous comprehensive post hoc analysis regarding the possible impact of duloxetine on self-rated suicidal ideation suggested the net effect of the drug on the suicidality item of the HDRS to be beneficial, but did not specifically address the issue of possible early effects or age [10]. The present results were similar to those previously obtained for SSRIs and venlafaxine; the mean rating of suicidality in duloxetine-treated subjects compared to those on placebo was hence significantly lower in subjects ≥25 years already from week one and onwards. In the younger age group, a corresponding superiority of duloxetine was also at hand from week one and onwards, but never reached the level of statistical significance; when interpreting this difference between the two age groups, it should, however, be noted that the number of subjects aged 18–24 was much lower (*n* = 257) than that of the older subjects (*n* = 3318). While these data do not exclude the possibility that duloxetine may exert a paradoxical suicide-provoking effect in susceptible individuals, they lend no support for the assumption that the net effect of the drug on suicidality, at the first week of treatment or at endpoint, be harmful, either in those below or above the age of 25. Since no children or adolescents were included, our data, however, permit no conclusions with respect to the possible effect of duloxetine on suicidality in these age groups.

Duloxetine displaying affinity not only for the transporter of serotonin, but also for that of noradrenaline [7], has prompted previous writers to suggest that this drug displays a somewhat different response profile as compared to that of the SSRIs. In line with this, a previous post hoc analysis from Mallinckrodt and co-workers, based on six trials comprising a duloxetine arm, an SSRI arm and placebo, suggested duloxetine to outperform the SSRIs with respect to a number of items (work and activities, psychomotor retardation, genital symptoms and hypochondriasis) [36]. To some extent, the interpretation of the outcome of these head-to-head comparisons is, however, marred by the fact that the SSRI dose applied in three of them, as acknowledged by the authors, may have been suboptimal [37, 38]. We hence used a different path to explore the same issue, i.e. to compare the relative item response profile (after adjustment for overall response) of the duloxetine-treated patients included in the dataset on which this paper is based with that of the SSRI-treated patients included in the development programs for citalopram, paroxetine and sertraline (*n* = 8262) [5]. This analysis revealed a strikingly similar response profile for the SSRIs and duloxetine, respectively, hence arguing against any major differences between these two treatments. While it should be underlined that adequately powered head-to-head trials using equipotent doses are required to definitely settle the issue of possible differences in efficacy or effect profile between two treatments, this lack of apparent differences in response profile is somewhat surprising; since duloxetine but not the SSRIs is reported to inhibit the noradrenaline transporter (NET), certain dissimilarities in clinical response profile might indeed have been expected. One possible explanation to this similarity of SSRIs and an SNRI, respectively, could be that the influence of duloxetine on NET in the human brain at the dosage used to treat depression may be lower than usually assumed; recent positron emission tomography studies thus indicate that duloxetine at clinically relevant dosage blocks less than 40% of NET but around 80% of the serotonin transporter (SERT) [39]. It is also possible that the contribution of additional NET inhibition to an antidepressant response obtained by SERT inhibitors has been overrated, and/or that the response pattern observed reflects the nature of the disorder rather than of the given treatment, i.e. that a similar response profile should be expected for any effective antidepressant regimen when compared to placebo. Similar studies comparing the response profile of SSRIs and duloxetine with that of antidepressants not influencing SERT or NET might shed further light on this issue. Finally, when interpreting the present data, it should be considered that there may indeed exist differences between duloxetine and SSRIs that are not captured by any of the HDRS-17 items; duloxetine has, for example, been attributed superior efficacy for the treatment of pain [40], which is an effect that is not necessarily reflected by the HDRS-17.

The present analysis differs from the majority (though not all) of previous meta-analyses in the antidepressant field by being conducted on patient- rather than trial-level, and by analysing individual symptoms rather than the sum score of several items. We believe that such an approach, which is in line with the RDoC focus on symptoms and dimensions rather than diagnoses [41], to be particularly useful when analysing a condition such as depression, where patients differ considerably with respect to symptom profile at baseline. Of note is that a number of widely spread assumptions regarding the antidepressant drugs, such as their symptom-reducing effect being secondary to side effects [9], or the antidepressant effect being restricted to those with severe depression [1, 8], were based on trial-level, HDRS-17-sum-based meta-analyses, which have, however, now been rebutted by analyses conducted on the patient level [22] and/or being item-based [24, 42] (including the present one).

To conclude, item-based analyses of duloxetine trials revealed this drug to exert a robust reduction in depressed mood and other core symptoms of depression that, for several of these, was significant already after 1 week of treatment. The effect of duloxetine was not restricted to those with high HDRS-17-sum at baseline and not secondary to side effects; moreover, no support for a suicide-provoking effect was obtained. Comparisons of the effect profiles of duloxetine and SSRIs, respectively, revealed a strikingly similar picture.

## Funding and disclosure

The study was supported by the Swedish Medical Research Council, AFA Insurance, the Swedish Brain Foundation and by a grant from the Swedish state under the agreement between the Swedish government and the county councils (ALF). FH has received speaker’s fees from Servier and H Lundbeck. EE has been on advisory boards and/or received speaker’s honoraria and/or research grants from Eli Lilly, Servier, GSK, H Lundbeck and Janssen Cilag. The remaining authors declare no competing interests.

## Supplementary information

Supplementary material
