# Supplementary material for: Item-based analysis of the effects of duloxetine in depression: a patient-level post hoc study
Source: Neuropsychopharmacology. 2019 Sep 14;45(3):553–60. doi: 10.1038/s41386-019-0523-4 (PMC6969189; doi:10.1038/s41386-019-0523-4)
Supplement: Supplementary file 1 — Supplementary material [file 41386_2019_523_MOESM1_ESM.pdf]

**Item-based analysis of the effects of duloxetine in depression: a patient-level post hoc study: Supplementary information**

*Supplementary Results Tables and Figures*

**Table S1:** Included trials.

**Table S2:** Baseline means and frequencies for HDRS-6, non-HDRS-6 and individual items in patients scoring ≤18 points and ≥27 points with respect to HDRS-17-sum at baseline, respectively.

**Table S3:** Baseline means, effect sizes and p-values for HDRS-17-sum and items 1 and 3 including low-dose trials HMAH and HMAI (sensitivity analysis).

**Table S4:** Baseline means, effect sizes and p-values for HDRS-17-sum and individual items including patients scoring <15 with respect to HDRS-17-sum at baseline (sensitivity analysis).

**Figure S1:** Effect sizes for HDRS-6, non-HDRS-6 and individual items in patients scoring ≤18 (a) and ≥27 (b) with respect to HDRS-17-sum at baseline, respectively.

**Figure S2:** Estimated endpoint means for the depressed mood item in duloxetine-treated subjects with mild, moderate and severe initial adverse events, respectively.

**Figure S3:** Estimated endpoint means and effect sizes for HDRS-17-sum in duloxetine-treated subjects with or without adverse events versus placebo-treated subjects (sensitivity analysis).

| | Group | N | N females | N completers (week 8) | Trial duration (acute phase) | *Comments* |
|---|---|---|---|---|---|---|
| *HMAQa* | DLX | 56 | 36 | 33 | 8 weeks | Forced titration -> 120 mg |
| | FLX 20 mg | 28 | 16 | 18 | | |
| | PLA | 57 | 38 | 32 | | |
| *HMAQb* | DLX | 64 | 43 | 46 | 8 weeks | Forced titration -> 120 mg |
| | FLX 20 mg | 32 | 19 | 20 | | |
| | PLA | 61 | 38 | 35 | | |
| *HMATa* | DLX 40 mg | 69 | 47 | 49 | 8 weeks | |
| | DLX 80 mg | 64 | 39 | 48 | | |
| | PRX 20 mg | 64 | 34 | 42 | | |
| | PLA | 67 | 45 | 47 | | |
| *HMATb* | DLX 40 mg | 65 | 36 | 46 | 8 weeks | |
| | DLX 80 mg | 73 | 49 | 46 | | |
| | PRX 20 mg | 60 | 44 | 38 | | |
| | PLA | 59 | 39 | 38 | | |
| *HMAYa* | DLX 80 mg | 90 | 68 | 81 | 8 weeks | |
| | DLX 120 mg | 90 | 68 | 82 | | |
| | PRX 20 mg | 80 | 54 | 70 | | |
| | PLA | 86 | 64 | 71 | | |
| *HMAYb* | DLX 80 mg | 92 | 61 | 84 | 8 weeks | |
| | DLX 120 mg | 99 | 74 | 88 | | |
| | PRX 20 mg | 94 | 66 | 86 | | |
| | PLA | 93 | 60 | 87 | | |
| *HMBHa* | DLX 60 mg | 123 | 80 | 85 | 9 weeks | |
| | PLA | 122 | 83 | 90 | | |
| *HMBHb* | DLX 60 mg | 128 | 84 | 86 | 9 weeks | |
| | PLA | 139 | 99 | 90 | | |
| *HMBV* | DLX 60 mg | 173 | 107 | 138 | 8 weeks | Elderly only |
| | PLA | 84 | 50 | 66 | | |
| *HMCB* | DLX 60 mg | 141 | 96 | 96 | 7 weeks | Brief Pain Inventory rating of at least 2 points for inclusion |
| | PLA | 141 | 87 | 102 | | |
| *HMCR* | DLX 60 mg | 204 | 129 | 151 | 8 weeks | |
| | ESC 10 mg | 202 | 145 | 164 | | |
| | PLA | 96 | 62 | 68 | | |

| | | | | | | |
|---|---|---|---|---|---|---|
| *HMFA* | DLX 60 mg | 190 | 124 | 156 | 12 weeks | Elderly only |
| | PLA | 90 | 53 | 65 | | |
| *HMFS* | DLX 60 mg | 509 | 316 | 428 | 8 weeks | |
| | PLA | 250 | 160 | 213 | | |
| *HMAH* | DLX 20 mg | 89 | 53 | 57 | 10 weeks | Phase II, low dosages |
| | PLA | 88 | 49 | 61 | | |
| *HMAI* | DLX 5 mg | 130 | 87 | 79 | 8 weeks | Phase II, low dosages |
| | DLX 10 mg | 129 | 83 | 95 | | |
| | DLX 20 mg | 131 | 84 | 89 | | |
| | CLP 150 mg | 132 | 98 | 83 | | |
| | PLA | 126 | 84 | 79 | | |

**Table S1: Included trials.** N = number of included subjects, DLX = duloxetine, FLX = fluoxetine, PLA = placebo, PRX = paroxetine, ESC = escitalopram, CLP = clomipramine.

| | HDRS-17-sum ≤18 (n=1 436) | | | HDRS-17-sum ≥27 (n=356) | | |
|---|---|---|---|---|---|---|
| **Measure of efficacy (scoring range)** | **Mean** | ***s.d.*** | **Frequency (score ≥ 1)** | **Mean** | ***s.d.*** | **Frequency (score ≥ 1)** |
| **HDRS-17-sum** | 14.9 | 3.1 | N/A | 29.0 | 2.1 | N/A |
| **HDRS-6-sum** | 8.7 | 2.6 | N/A | 14.2 | 1.5 | N/A |
| **non-HDRS-6-sum** | 6.2 | 2.1 | N/A | 14.8 | 2.1 | N/A |
| | | | | | | |
| **HDRS-6 items** | | | | | | |
| *Depressed mood (0-4)* | 2.1 | 0.9 | 95% | 3.1 | 0.5 | 100% |
| *Feelings of guilt (0-4)* | 1.1 | 0.8 | 73% | 2.1 | 0.7 | 97% |
| *Work & activities (0-4)* | 2.1 | 0.9 | 94% | 3.1 | 0.5 | 100% |
| *Retardation (0-4)* | 0.6 | 0.7 | 52% | 1.4 | 0.8 | 87% |
| *Anxiety, psychic (0-4)* | 1.4 | 0.8 | 86% | 2.7 | 0.7 | 99% |
| *Somatic symptoms, general (0-2)* | 1.3 | 0.7 | 87% | 1.8 | 0.4 | 98% |
| **non-HDRS-6 items** | | | | | | |
| *Suicidal ideation (0-4)* | 0.3 | 0.6 | 26% | 1.2 | 0.9 | 73% |
| *Insomnia, early (0-2)* | 0.7 | 0.8 | 51% | 1.8 | 0.5 | 95% |
| *Insomnia, middle (0-2)* | 0.9 | 0.8 | 62% | 1.8 | 0.5 | 96% |
| *Insomnia, late (0-2)* | 0.7 | 0.8 | 50% | 1.5 | 0.7 | 89% |
| *Agitation (0-4)* | 0.6 | 0.7 | 52% | 1.4 | 1.0 | 87% |
| *Anxiety, somatic (0-4)* | 1.0 | 0.8 | 74% | 2.1 | 0.7 | 98% |
| *Somatic symptoms, gastrointestinal (0-2)* | 0.3 | 0.5 | 31% | 1.1 | 0.7 | 75% |
| *Genital symptoms (0-2)* | 0.9 | 0.8 | 60% | 1.7 | 0.6 | 91% |
| *Hypochondriasis (0-4)* | 0.6 | 0.7 | 44% | 1.6 | 1.0 | 85% |
| *Loss of weight (0-2)* | 0.1 | 0.3 | 7% | 0.6 | 0.8 | 39% |
| *Insight (0-2)* | 0.1 | 0.3 | 9% | 0.2 | 0.4 | 18% |

**Table S2: Baseline means and frequencies for HDRS-17-sum, HDRS-6-sum, non-HDRS-6-sum and individual items in patients scoring ≤18 points and ≥ 27 points at baseline, respectively.**
*s.d.* = standard deviation.

| **Measure of efficacy (scoring range)** | **Baseline mean** | ***s.d.*** | **ES** | **p-value** | **ES** | **p-value** | **ES** | **p-value** |
|---|---|---|---|---|---|---|---|---|
| | | | *Week 1* | | *Week 6* | | *Week 8* | |
| *HDRS-17-sum* | 21.5 | 4.0 | 0.04 | 0.25 | 0.33 | <0.001 | 0.36 | <0.001 |
| *Depressed mood (0-4)* | 2.7 | 0.6 | 0.19 | <0.001 | 0.42 | <0.001 | 0.41 | <0.001 |
| *Suicidal ideation (0-4)* | 0.7 | 0.8 | 0.12 | <0.001 | 0.21 | <0.001 | 0.23 | <0.001 |

**Table S3: Baseline means, effect sizes and p-values for HDRS-17-sum and items 1 and 3 including low-dose trials HMAH and HMAI.**
Placebo *n* = 1 559; duloxetine *n* = 2 709. *s.d.* = standard deviation; *ES* = effect size.

| **Measure of efficacy (scoring range)** | **Baseline mean** | ***s.d.*** | **ES** | **p-value** | **ES** | **p-value** | **ES** | **p-value** |
|---|---|---|---|---|---|---|---|---|
| | | | *Week 1* | | *Week 6* | | *Week 8* | |
| *HDRS-17-sum* | 20.1 | 5.0 | 0.04 | 0.26 | 0.34 | <0.001 | 0.34 | <0.001 |
| *Depressed mood (0-4)* | 2.6 | 0.8 | 0.20 | <0.001 | 0.44 | <0.001 | 0.41 | <0.001 |
| *Feelings of guilt (0-4)* | 1.5 | 0.9 | 0.10 | 0.001 | 0.26 | <0.001 | 0.26 | <0.001 |
| *Suicidal ideation (0-4)* | 0.6 | 0.7 | 0.15 | <0.001 | 0.22 | <0.001 | 0.23 | <0.001 |
| *Insomnia, early (0-2)* | 1.1 | 0.9 | 0.05 | 0.17 | 0.02 | 0.63 | 0.07 | 0.05 |
| *Insomnia, middle (0-2)* | 1.2 | 0.8 | -0.11 | 0.001 | 0.01 | 0.85 | 0.02 | 0.64 |
| *Insomnia, late (0-2)* | 1.0 | 0.8 | -0.04 | 0.27 | 0.17 | <0.001 | 0.13 | 0.004 |
| *Work & activities (0-4)* | 2.5 | 0.8 | 0.03 | 0.30 | 0.26 | <0.001 | 0.29 | <0.001 |
| *Retardation (0-4)* | 0.9 | 0.8 | 0.03 | 0.43 | 0.26 | <0.001 | 0.19 | <0.001 |
| *Agitation (0-4)* | 0.9 | 0.9 | 0.08 | 0.01 | 0.12 | 0.004 | 0.10 | 0.01 |
| *Anxiety, psychic (0-4)* | 1.9 | 0.9 | 0.22 | <0.001 | 0.40 | <0.001 | 0.39 | <0.001 |
| *Anxiety, somatic (0-4)* | 1.4 | 0.8 | -0.06 | 0.09 | 0.07 | 0.08 | 0.10 | 0.01 |
| *Somatic symptoms, gastrointestinal (0-2)* | 0.6 | 0.6 | -0.30 | <0.001 | 0.04 | 0.38 | 0.06 | 0.08 |
| *Somatic symptoms, general (0-2)* | 1.5 | 0.6 | -0.03 | 0.38 | 0.23 | <0.001 | 0.18 | <0.001 |
| *Genital symptoms (0-2)* | 1.1 | 0.8 | -0.12 | <0.001 | 0.05 | 0.23 | 0.09 | 0.01 |
| *Hypochondriasis (0-4)* | 0.9 | 0.9 | 0.04 | 0.22 | 0.22 | <0.001 | 0.22 | <0.001 |
| *Loss of weight (0-2)* | 0.2 | 0.5 | -0.21 | <0.001 | -0.04 | 0.36 | -0.04 | 0.26 |
| *Insight (0-2)* | 0.1 | 0.4 | -0.06 | 0.09 | 0.00 | 0.95 | 0.04 | 0.27 |

**Table S4: Baseline means, effect sizes and p-values for HDRS-17-sum and individual items including patients scoring <15 at baseline.**
Placebo: *n* = 1 750; duloxetine: *n* = 3 006. *s.d.* = standard deviation; *ES* = effect size.

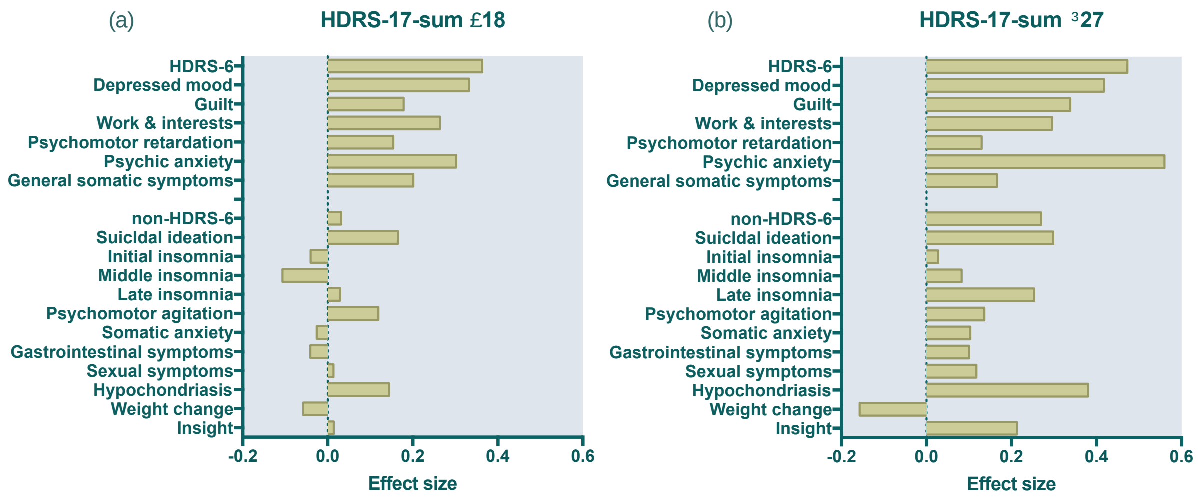


**Figure S1: Effect sizes for HDRS-6, non-HDRS-6 and individual items (a) patients scoring 18 points and lower, (b) 27 points and higher at baseline on the HDRS-17.**
(a) Placebo *n* = 541, duloxetine *n* = 897 (b) Placebo *n* = 129, duloxetine *n* = 227. Effect sizes (a) 0.36, 0.33, 0.18, 0.26, 0.15, 0.30, 0.20, 0.03, 0.17, -0.04, -0.11, 0.03, 0.12, -0.03, -0.04, 0.01, 0.14, -0.06, 0.01 (b) 0.47, 0.42, 0.34, 0.30, 0.13, 0.56, 0.17, 0.27, 0.30, 0.03, 0.08, 0.25, 0.14, 0.10, 0.10, 0.12, 0.38, -0.16, 0.21.

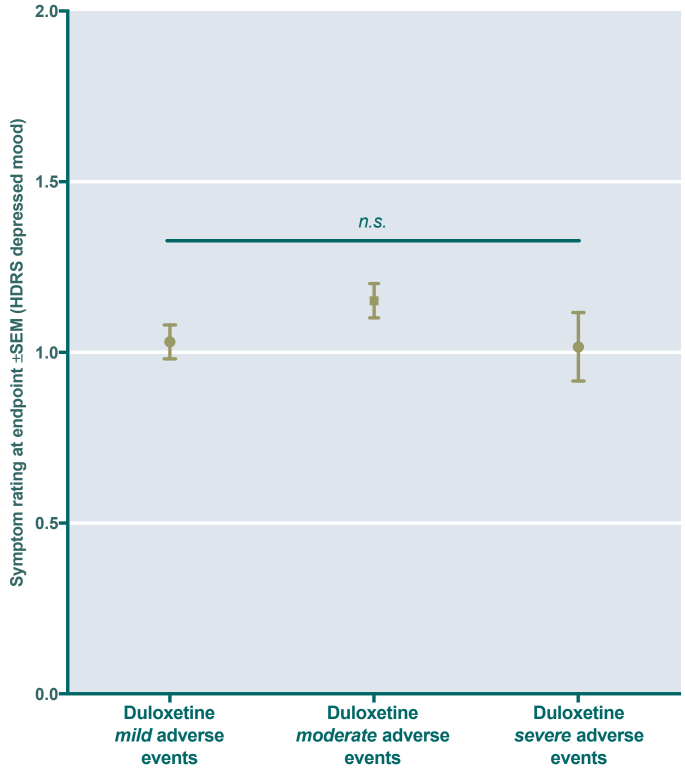


**Figure S2: Estimated endpoint means for the depressed mood item in duloxetine-treated subjects with mild, moderate and severe initial adverse events, respectively.**
Duloxetine with mild adverse events *n* = 477, moderate *n* = 452, severe *n* = 100. Omnibus analysis of covariance p-value = 0.14. *n.s.* = non-significant.

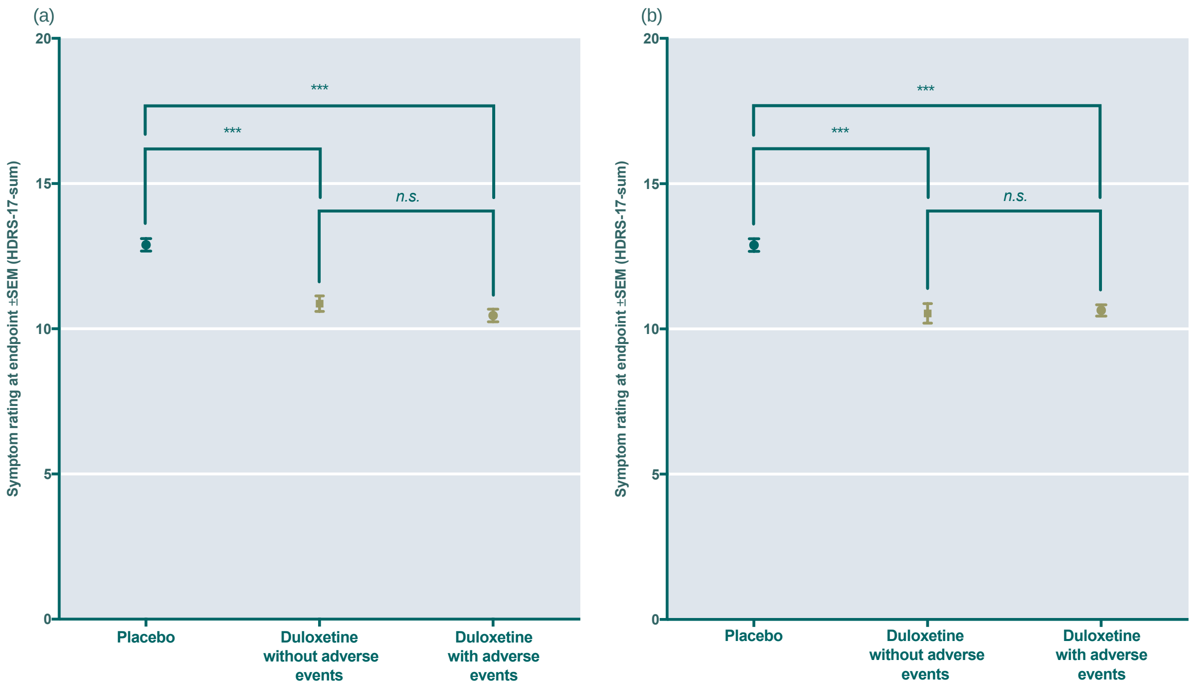


**Figure S3: Estimated endpoint means and effect sizes for HDRS-17-sum in placebo-treated subjects versus duloxetine-treated subjects with or without adverse events only counting initial adverse events (week 1-2) (a) and counting any adverse event throughout the trial (b).**
(a) Placebo: *n* = 998, duloxetine without adverse events: *n* = 713, duloxetine with adverse events: *n* = 1 029; (b) Placebo: *n* = 998, duloxetine without adverse events: *n* = 449, duloxetine with adverse events: *n* = 1 293. Effect sizes: (a) duloxetine with adverse events vs. placebo 0.38, duloxetine without adverse events vs. placebo 0.32, duloxetine with adverse events vs. duloxetine without adverse events 0.06 (b) duloxetine with adverse events vs. placebo 0.35, duloxetine without adverse events vs. placebo 0.37, duloxetine with adverse events vs. duloxetine without adverse events -0.02. *n.s.* = non-significant ($p = 0.22$ (a), $p = 0.78$ (b)), *** = $p < 0.001$.
